# Protein-dependent Membrane Interaction of A Partially Disordered Protein Complex with Oleic Acid: Implications for Cancer Lipidomics

**DOI:** 10.1038/srep35015

**Published:** 2016-10-12

**Authors:** Arunima Chaudhuri, Xavier Prasanna, Priyanka Agiru, Hirak Chakraborty, Anna Rydström, James C. S. Ho, Catharina Svanborg, Durba Sengupta, Amitabha Chattopadhyay

**Affiliations:** 1 CSIR-Centre for Cellular and Molecular Biology, Uppal Road, Hyderabad 500 007, India; 2 CSIR-National Chemical Laboratory, Pune 411 008, India; 3 Department of Microbiology, Immunology and Glycobiology (MIG), Institute of Laboratory Medicine, Lund University, Lund, Sweden; 4 Centre for Biomimetic Sensor Science, School of Materials Science & Engineering, Nanyang Technological University, 637553 Singapore

## Abstract

Bovine α-lactalbumin (BLA) forms cytotoxic complexes with oleic acid (OA) that perturbs tumor cell membranes, but molecular determinants of these membrane-interactions remain poorly understood. Here, we aim to obtain molecular insights into the interaction of BLA/BLA-OA complex with model membranes. We characterized the folding state of BLA-OA complex using tryptophan fluorescence and resolved residue-specific interactions of BLA with OA using molecular dynamics simulation. We integrated membrane-binding data using a voltage-sensitive probe and molecular dynamics (MD) to demonstrate the preferential interaction of the BLA-OA complex with negatively charged membranes. We identified amino acid residues of BLA and BLA-OA complex as determinants of these membrane interactions using MD, functionally corroborated by uptake of the corresponding α-LA peptides across tumor cell membranes. The results suggest that the α-LA component of these cytotoxic complexes confers specificity for tumor cell membranes through protein interactions that are maintained even in the lipid complex, in the presence of OA.

Many soluble proteins in partially disordered states are known to interact with lipid membranes, and such interactions play crucial roles in several cellular processes^1^,^2^,^3^. The small acidic Ca^2+^-binding protein (mol. wt. 14.2 kDa), bovine α-lactalbumin (BLA), is a prototype for such partially disordered proteins that selectively interact with membranes of certain compositions, in a narrow window of pH, temperature and ionic strengths^4^,^5^,^6^. It is present in milk and functions as a specificity modifier of galactosyltransferase to aid lactose synthesis in lactating mammals^7^,^8^. It has several partially unfolded intermediate states, collectively known as molten globules, formed under various conditions such as acidic pH by removal of the strongly bound Ca^2+^ ion^9^,^10^. Due to its conformational plasticity, the milk protein α-Lactalbumin (α-LA) represents an excellent prototype for monitoring membrane interactions of partially disordered proteins.

Interestingly, α-LA forms complexes with oleic acid (OA), which have been shown to posses potent tumoricidal and anti-microbial properties^11^,^12^,^13^,^14^,^15^,^16^,^17^,^18^,^19^,^20^,^21^,^22^. In view of the tumoricidal activity of the complex of BLA with OA, it has been given the acronym BAMLET (bovine α-lactalbumin made lethal to tumor cells^12^). The complex was originally identified with the human homolog of BLA, and given the acronym HAMLET^23^,^24^,^25^. The biophysical properties of BAMLET^21^,^22^,^23^,^24^,^25^ and HAMLET^21^,^26^,^27^,^28^,^29^,^30^,^31^,^32^,^33^ and their cytotoxicity^11^,^12^,^13^,^14^,^15^,^21^ has been explored in some detail.

The efficacy of HAMLET as a selective killer of tumor cells has been documented *in vitro* and *in vivo* in several animal models and clinical studies^34^,^35^,^36^,^37^,^38^. Membrane interactions are essential to trigger tumor cell death and direct effects on membranes are the first step in this process^39^. In vesicular membranes consisting of egg yolk or soybean lipids, HAMLET caused membrane elongation and altered fluidity^40^. In giant unilamellar phosphocholine vesicles, rapid membrane insertion of HAMLET, triggered membrane tubulation^40^. However, these studies have not explored effects of the protein-OA complex on membrane lipids in detail or interactions of defined protein domains with the lipid bilayer. In this manuscript, we have explored the membrane interactions of α-LA-OA complex, which is a crucial defining factor responsible for its anti-microbial and tumoricidal effects.

In this study, we resolved residue-specific interactions of BLA with OA using molecular dynamics (MD) simulation. In addition, by using the voltage-sensitive membrane bound fluorescent membrane probe, 4-(2-(6-(dioctylamino)-2-naphthalenyl)ethenyl)-1-(3-sulfopropyl)-pyridinium inner salt (di-8-ANEPPS) combined with coarse-grain (CG) MD simulations, we show that there is a preferential interaction of the BLA-OA complex with negatively charged lipids. Similar results were obtained in MD simulations for BLA alone and residues involved in the interactions were mapped, suggesting that the specificity arises from the molecular interactions of the protein. We also obtained evidence that the identified residues interact with tumor cell membranes, as data on uptake of the peptide fragments mapping the entire sequence of α-LA provided experimental validation to the membrane interacting residues predicted from simulations. A novel observation was the remarkable clustering of negatively charged lipids in the vicinity of the membrane-bound BLA/BLA-OA complex. Our integrated approach, combining experiments with simulations, to monitor the interaction of BLA-OA complex with membranes, provide holistic insights into lipid-protein interaction governing the affinity of BLA-OA for membranes.

## Results

### Characterization of the BLA-OA complex

**Figure 1:**
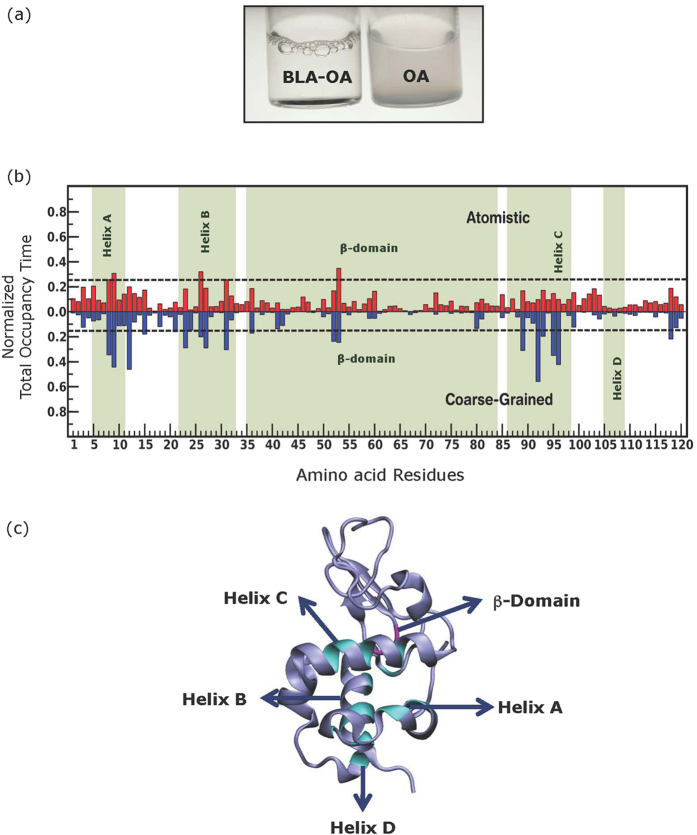
(**a**) Images of BLA-OA and OA solutions. OA solution (pH 7.4) is highly turbid (absorbance at 400 nm ~0.5). Addition of BLA significantly decreases the turbidity (absorbance at 400 nm ~0.05). (**b**) Total occupancy time of a single OA around the residues of BLA. The total occupancy time of a single OA around the residues of BLA normalized over the total bound period and averaged over ten atomistic (upper panel) and coarse-grained (lower panel) simulations are shown. The dotted line represents mean ± 2 S.D. The mean and SD for atomistic and CG are 0.09 ± 0.075 and 0.07 ± 0.05, respectively. The corresponding values of SE are 0.006 and 0.005, respectively. The residues on and above the dotted line (both in the upper and lower panels) represent the most probable residues interacting with a single OA. (**c**) Schematic representation of BLA (ice blue) and the residues having most probable interaction with oleic acid in α- (cyan) and β- (magenta) domains are highlighted. See Experimental Section for further details. See Supplementary Figure S2 for representative snapshots showing few of the sites sampled by a single OA molecules on BLA.

BLA is intrinsically fluorescent due to the presence of four tryptophans (at positions 26, 60, 104 and 118)^46^ and these suitably positioned tryptophans in BLA act as convenient probes to explore its conformation and dynamics under various conditions^5^,^9^,^10^. The BLA-OA complex was prepared by a modification of the mixing method^18^,^19^,^20^,^21^,^26^ (see Experimental Section for details). We verified the formation of the complex by turbidity analysis, detecting a significant reduction in the opacity of the OA sample upon addition of BLA (Fig. 1a), a characteristic feature earlier observed upon formation of protein-lipid complexes^18^,^26^. A red shifted emission maximum at 340 nm was detected for the tryptophans in the BLA-OA complex, compared to an emission maximum of 337 nm in BLA, when excited at 295 nm (Figure S1a). The red shift might reflect enhanced water penetration as a result of loss of the tertiary interaction (see CD results) upon OA binding.

An increase in tryptophan fluorescence intensity was recorded in BLA upon formation of BLA-OA complex relative to BLA alone (~1.2 fold), (Figure S1a). This increase in fluorescence intensity was corroborated by an increase in the fluorescence lifetime of BLA-OA complex (3.9 ns) relative to BLA (3.2 ns, Figure S1b), when excited at 295 nm. The increase in fluorescence intensity could be attributed to the release of quenching around the tryptophan residues associated with partial disordering. The results suggest that complex formation with OA allows the protein to reach a more flexible tertiary (molten globule) state, leading to a further increase in fluorescence intensity relative to *apo*-BLA.

### Protein folding

To confirm this hypothesis, we analyzed the CD spectral characteristics of the secondary and tertiary structures of *apo*-BLA-OA relative to *apo*-BLA (Figure S1c,d). A considerable loss of tertiary structure was detected by near-UV CD spectroscopy, relative to BLA in solution (Figure S1c,d). However, the secondary structure of the BLA-OA complex was intact, as shown by the far-UV CD spectra, indicating that BLA is a molten globule, which represents a milieu of conformations with various degrees of unfolding.

### Plasticity and dynamics of BLA-OA interactions

To monitor the interaction of *apo*-BLA with a single OA, we performed ten atomistic and coarse-grained simulations for a time period of 0.5 and 1 μs, respectively. The normalized total occupancy time of a single OA with each residue of BLA was calculated from atomistic and CG simulation (Fig. 1b, referred to as occupancy time). The mean occupancy time obtained from atomistic and CG simulations were 0.09 and 0.07 μs, respectively, with no single site of high occupancy for OA around the residues of BLA. The low mean value indicates that there is an inherent plasticity in the residue level interaction of OA with BLA and that residues from almost all segments of the protein may interact with OA (see Figure S2 for snapshots of such interaction). Residues with a probability score ≥ mean + 2 S.D. were mapped to the *apo*-crystal structure of BLA^46^. Few residues could be identified in helices A, B and C, 3_10_, loop regions of the α-domain and in the β-domain (Table S1).

A highly dynamic interaction of the fatty acyl chain of OA with various BLA residues was demonstrated by the multiple trajectories traced by a single oleic acid, during 100 ns time regime of simulation (see Figure S2 in Supporting Information for snapshots). Interestingly, a similar pattern of residue level plasticity was detected by CG simulation monitoring the sequential binding of OA (see Table S1 and Figure S3 in Supporting Information for details). The results suggest that residues showing high occupancy around a single OA were conserved and remained interactive, following the sequential binding of six OA to BLA. The results of CG and atomistic simulations are also consistent with earlier observations based on experimental approaches such as NMR^33^, H-D exchange^30^, limited proteolysis^30^ and SAXS^32^ (see Table S1 in Supporting Information for details).

### BLA/BLA-OA complex interaction with membranes of different composition, monitored by dual ratiometric fluorescence read out from di-8-ANEPPS fluorescence

**Figure 2:**
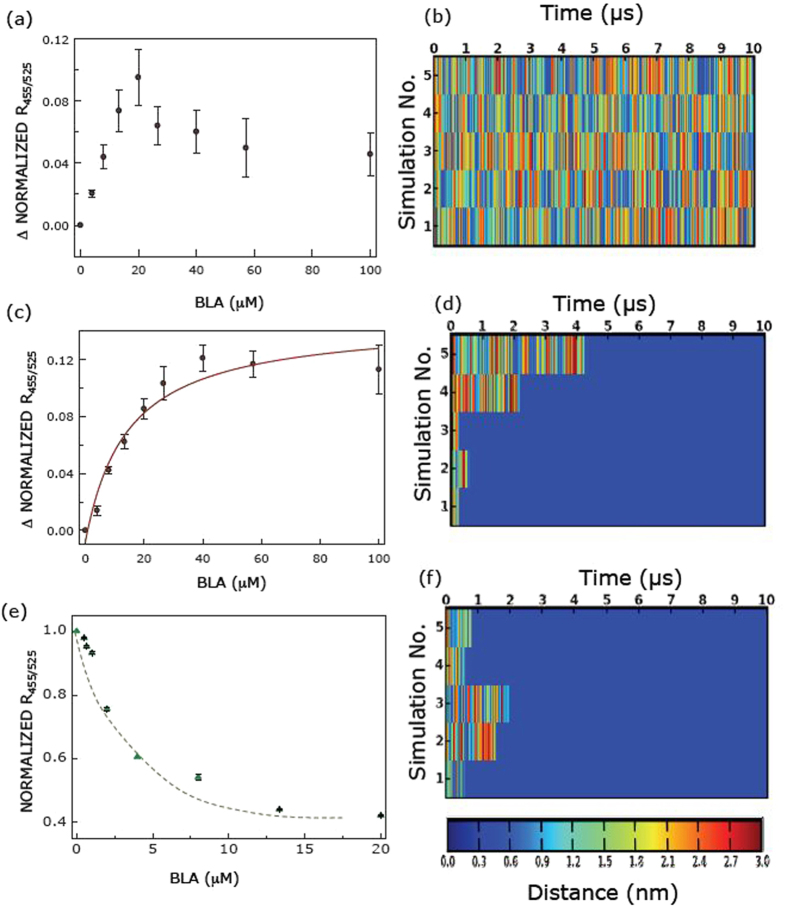
Differential interaction of BLA in BLA-OA complex with membranes using simulation and experiment. Membrane interaction of BLA in the complex monitored using the fluorescence of the potential-sensitive probe di-8-ANEPPS (**a**,**c**). The difference in normalized R_455/525_ (the normalized R_455/525_ for BLA-OA complex and OA suspension additions with membranes of various compositions are shown in Figure S4) between *apo*-BLA-OA complex and OA with increasing concentration of *apo*-BLA are shown for (**a**) POPC and (**c**) 30% POPG/70% POPC membranes. (**e**) Normalized change in fluorescence ratio (R_455/525_) of the excitation spectra of di-8-ANEPPS with increasing *apo*-BLA concentration are shown for comparison (data taken from ref. 5). (**b**,**d**) Time course of interaction of BLA-OA complex with bilayers of various composition. The minimum distance between BLA and lipid (defined as the closest distance of approach between CG beads of BLA and lipid) during the course of five simulations are plotted for (**b**) POPC and (**d**) 30% POPG/70% POPC membranes. The color bar below refers to the scale for minimum distance of interaction. Blue regions represent close contacts relative to red regions which indicate larger distance between BLA and the bilayer. (**f**) Time course of interaction of BLA with PC/PG bilayers using CG simulation is shown for comparison with BLA-OA complex-membrane interaction shown in (**d**). The shorter distances of minimum interaction is color coded blue (refer to the scale bar below **b,d,f**). See Experimental Section for further details.

The membrane interaction of BLA is regulated by variables such as membrane composition, pH and curvature^4^,^5^,^6^. To delineate the effect of BLA in the BLA-OA complex on membranes of varying composition, we utilized the membrane-bound voltage-sensitive probe, di-8-ANEPPS in the dual ratiometric approach^5^,^6^,^47^, *i.e.*, monitoring the ratio of fluorescence intensity at 455 and 525 nm from the excitation spectra while fixing the emission wavelength at 670 nm. OA and BLA-OA decreased the normalized fluorescence ratio R_455/525_ (Figure S4). We further calculated the difference in the normalized ratio (ΔNormalized R_455/525_) of the membrane bound probe, obtained by subtracting R_455/525_ upon addition of the BLA-OA complex from that of OA alone, for the same concentration of OA. This analysis was performed with increasing concentrations of OA and BLA-OA complex (see Fig. 2a,c), based on the assumption that the change in the normalized fluorescence ratio induced by BLA and OA is additive in the BLA-OA complex.

In zwitterionic membranes composed of 1-palmitoyl-2-oleoyl-*sn*-glycero-3-phosphocholine (POPC), (Fig. 2a), the dose-dependent change in normalized ratio did not reach saturation, and therefore could not be fitted to any pre-existing binding model. Similar results were earlier observed for BLA binding to zwitterionic membranes (Figure S5b, data taken from ref. 5). Interestingly, the dose-dependent change in normalized ratio (ΔNormalized R_455/525_) in negatively charged membranes followed a hyperbolic curve. Data was analyzed according to single binding site model to yield apparent K_d_ of ~14.4 μM for BLA in BLA-OA complex (see Fig. 2c). These results suggest that BLA in the BLA-OA complex (see Fig. 2c) retains the specificity of binding to negatively charged membranes as is exhibited by BLA alone (see Fig. 2e).

### Simulations of BLA/BLA-OA complex interactions with membranes of different composition

The molecular determinants of binding to phosphatydylcholine (PC) and phosphatidylcholine/phosphatidylglycerol (PC/PG) membranes of the defined BLA-OA complex with 6 OA bound per BLA, were examined in a total of five CG simulations. The estimated minimum distance between BLA in BLA-OA complex and membrane was chosen as a measure of their interaction, using simulation carried out for a period of 10 μs (Fig. 2b,d). The results show that multiple events of binding and unbinding occur, without any extended time of stable binding (blue color in the plots) of BLA-OA complex to PC membranes (Fig. 2b). Similar results of multiple events of binding and unbinding also occur in the time-course of interaction of BLA with zwitterionic membranes (Figure S5a). In contrast, following an initial short period of multiple binding and unbinding events, an extended stable period of interaction (blue color in the plots) was established for the BLA-OA complex with PC/PG membranes (Fig. 2d). Interestingly, we observed a similar pattern of affinity in the minimum distance plots representing membrane interactions of BLA alone with negatively charged membranes (Fig. 2f).

These results demonstrate that BLA and the BLA-OA complex interact with negatively charged bilayers, a conclusion supported by experiments and simulations. We suggest that the findings might be used as a model for lipid-protein interaction of partially disordered proteins in general, and α-lactalbumin in particular.

### Mapping the residues of BLA/BLA-OA complex interacting with lipids at various membrane depths

**Figure 3:**
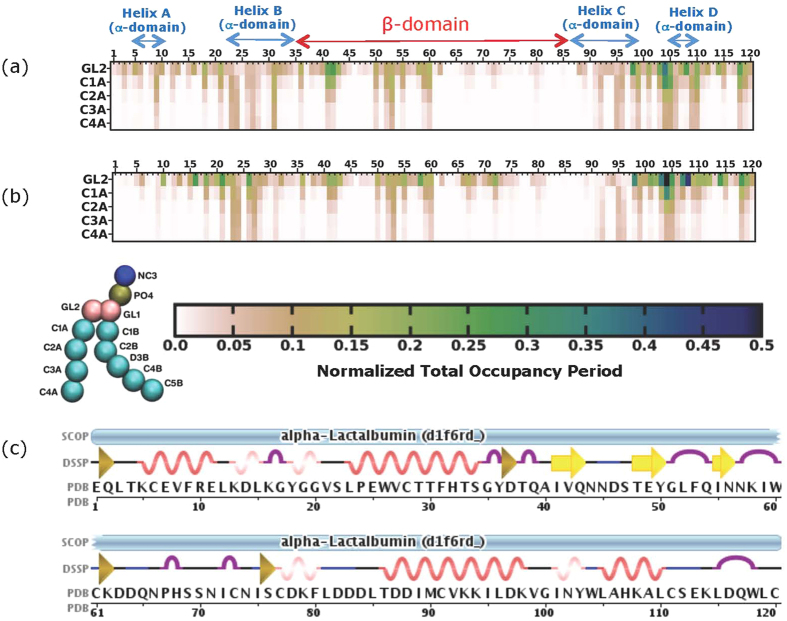
The time-averaged occupancy maps depicting interaction between BLA (**a**) and BLA-OA complex (**b**) with PC/PG lipid bilayers. The total occupancy times for BLA residues around CG beads representing the *sn*-2 glycerol (GL2) and palmitoyl chain (C1A, C2A, C3A and C4A) of lipids were calculated and normalized to the total membrane bound period. The values given in the figure represent the average over all simulations. A common scale bar for the contact distances and a schematic CG representation of POPG are shown at the bottom. (**c**) A schematic representation of the secondary structure of BLA is given (figure taken from RSCB; PDB ID: 1F6R). These secondary structural elements are also highlighted on the top of panel (**a**) for reference. See Experimental Section for further details. To see this figure in color, go online.

To visualize the residue-specific interactions governing the membrane binding of partially disordered proteins, we monitored the residue level interaction of BLA (Fig. 3a) and BLA-OA complex (Fig. 3b) with negatively charged membrane bilayers (70% POPC/30% 1-palmitoyl-2-oleoyl-*sn*-glycero-3-phosphatidylglycerol (POPG). The normalized lipid membrane occupancy of amino acids in BLA, alone or in complex with OA was determined along the bilayer normal (*i.e.*, along the membrane z-axis). The occupancy times around various regions of the palmitoyl chain was assessed and residues interacting with secondary structural elements were identified, based on the crystal structure of *apo*-BLA (wiring diagram of BLA shown in Fig. 3c). The residues present in close proximity of the lipid chains, in BLA alone and in complex with OA, were shown to lie predominantly in the α-domain and some in the β-domain. Particularly, residues in the C-terminal part of BLA, alone and in complex with OA, showed relatively high occupancy time around the *sn-*2 glycerol carbon (GL2) of lipids in negatively charged membrane bilayers (see Table S2 for details of the interacting residues). The shallowness of the interactions of the residues in BLA/BLA-OA complex around the different regions of the lipid chains with respect to the membrane bilayer z-axis was unexpected (see Tables S3 and S4 for details of the interacting residues).

The findings suggest that the association of BLA with OA does not preclude further interactions between the protein and negatively charged membranes and that a number of membrane interacting residues are conserved in BLA after the formation of BLA-OA complex. This probably indicates that BLA guides the specificity of interaction with membranes even after formation of BLA-OA complex. These results corroborate well with our experimental binding results, where we observed that both BLA and BLA-OA complex exhibit similar membrane binding specificities (ref. 6; Figs 2 and S5). However, there are subtle differences in the interaction patterns of BLA in complex with OA relative to BLA alone.

### Validation of the membrane interacting residues in BLA/BLA-OA complex using peptide uptake in tumor cells

**Figure 4:**
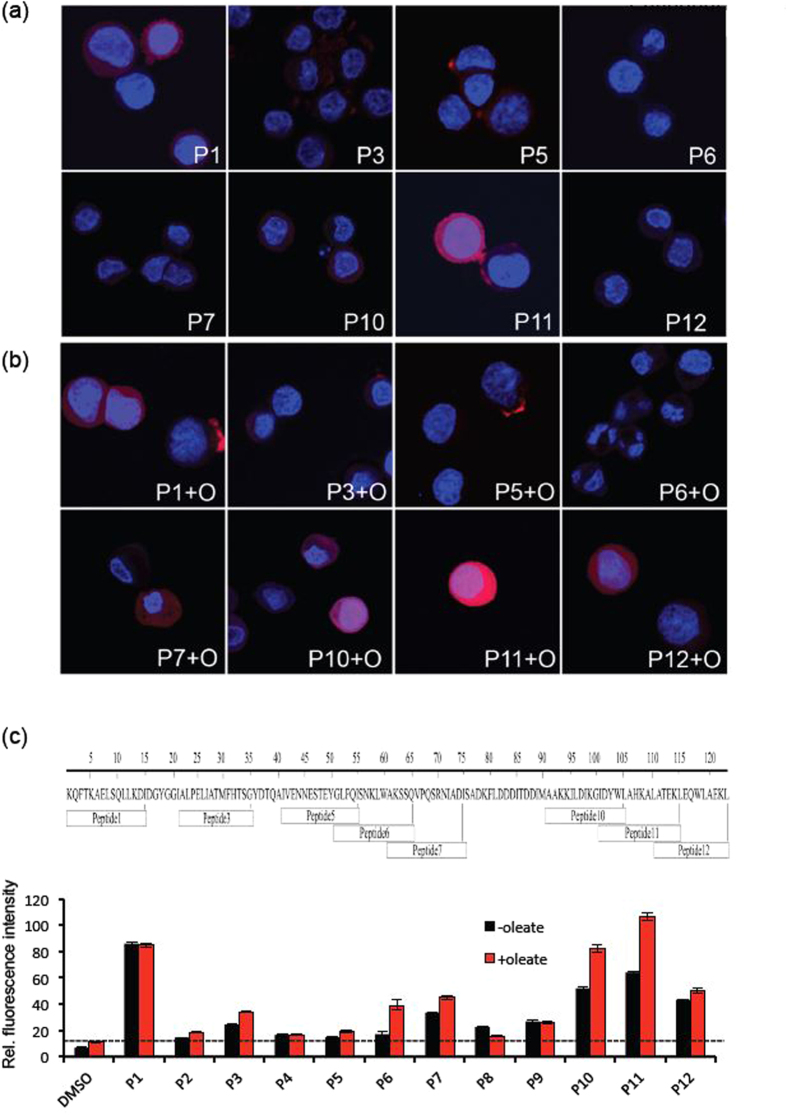
Internalization of specific α-lactalbumin peptides by tumor cells. (**a**) Internalization of biotinylated peptides (105 μM, red) by A549 lung carcinoma cells counterstained with Hoechst (blue) and examined by confocal microscopy. Peptides 1, 10 and 11 are more efficiently internalized as compared to other peptides in the absence of oleate. (**b**) The presence of oleate (175 μM, α-LA/OA ratio of 0.6 (mol/mol)) enhances the internalization of peptides 1, 10 and 11. Similarly, internalization of peptides 6, 7 and 12 are increased. (**c**) One-dimensional schematic representation of peptide sequences and the respective fluorescence quantification, summarizing the internalization efficiency for each peptide by tumor cells. Data adapted from ref. 32.

The pattern of membrane interaction based on *in vitro* experiments and simulations was compared to the uptake by tumor cells of synthetic human-α-LA peptides, 15 amino acids in length with a five amino acid overlap (Fig. 4)^32^. By quantitative confocal microscopy, we detected enhanced uptake of peptide fragments mapping the C-terminal (P-10, P11, P-12) region of α-LA. The uptake of these peptides was significantly enhanced in the presence of OA (p < 0.01), Fig. 4b). Minimal uptake of peptide 8 (residues 70–85) corresponded to the low occupancy in the simulation map. Enhanced uptake of peptides in the N-terminal- and β-domain (P3, P5, P6, P7) were also observed upon formation of complex, as predicted by simulations (see Fig. 3). These results provide a strong experimental validation to our observations from coarse-grained simulations shown in Fig. 3. These results reassert the self-consistency among the various experimental observations, *i.e.*, membrane binding studies using di-8-ANEPPS; molecular interaction monitored by CG simulation and cellular uptake assay explored using confocal microscopy.

### Effect of BLA/BLA-OA on bilayer properties

**Figure 5:**
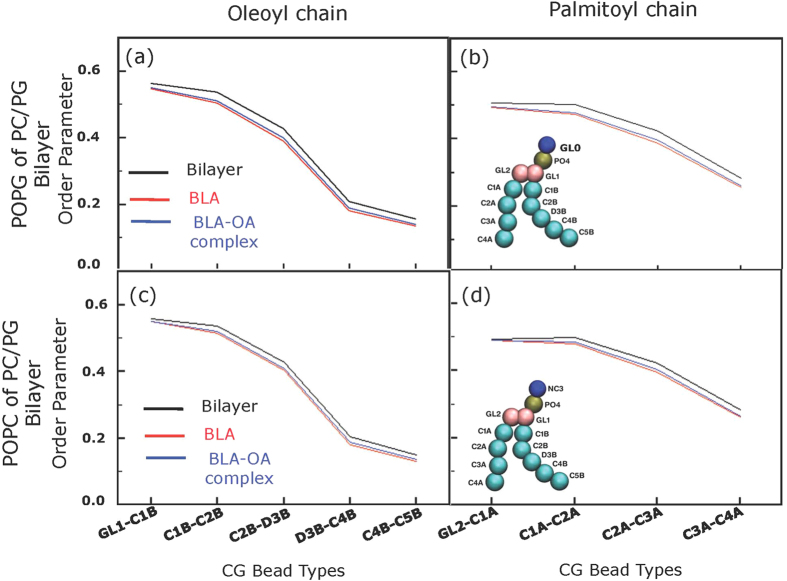
One-dimensional order parameter of POPG (**a**,**b**) and POPC (**c**,**d**) in PC/PG bilayer in the presence of BLA and BLA-OA complex. The order parameters for the oleoyl (**a**,**c**) and palmitoyl (**b**,**d**) chains in the presence of BLA (red line) and BLA-OA (blue line) are shown. The order parameter of the membrane alone is given in black as control. Schematic CG representation of POPG and POPC are given as insets of panels b and d. See Experimental Section for further details.

The one-dimensional order parameter for the acyl chains of lipids (*i.e.*, palmitoyl and oleoyl) in PC/PG bilayers decrease following the binding of BLA/BLA-OA complex (Fig. 5, see insets of Fig. 5b,d for schematic CG representation POPG and POPC, respectively). The one-dimensional order parameter was more strongly reduced in the POPG (Fig. 5a,b) component of the bilayer than in POPC (Fig. 5c,d). Importantly, no significant difference was observed in the order parameter of the acyl chains (*i.e.*, palmitoyl and oleoyl) between BLA and BLA-OA complex.

**Figure 6:**
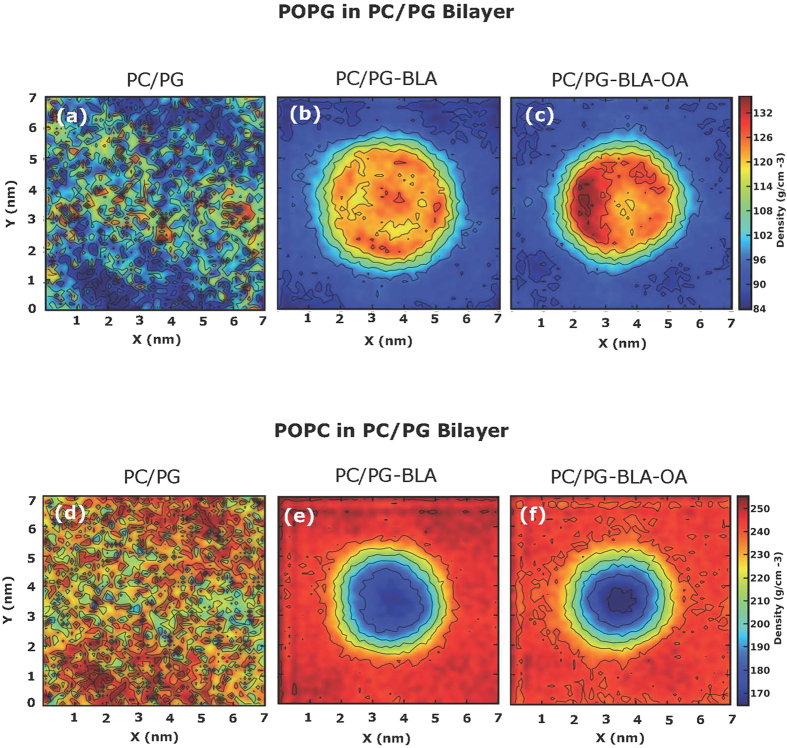
Two-dimensional representation of the individual lipid density in PC/PG bilayer in the absence (**a**,**d**); and presence of BLA (**b**,**e**) and BLA-OA complex (**c**,**f**). Density distribution of POPG (**a**–**c**) and POPC (**d**–**f**) in PC/PG bilayer are shown in the x-y planes. See Experimental Section for further details.

For the two-dimensional density distribution of different lipid components (*i.e.* POPC and POPG) following the stable binding of BLA and BLA-OA complex to the PC/PG bilayer, we observed a characteristic clustering of POPG around the site of protein binding in case of BLA/BLA-OA complex (see Fig. 6b,c). The clustering of POPG was concomitant with a low density of POPC at the site of protein binding in case of BLA/BLA-OA complex in this membrane system (see Fig. 6e,f). The control two-dimensional density distribution of POPG and POPC in the absence of BLA or BLA-OA are shown in Fig. 6a,d.

## Discussion

A number of studies have shown that partial unfolding is a crucial common feature of membrane interacting proteins and to guide the translocation of soluble proteins across membranes, especially in tumor cells^1^,^48^. Using dual-ratiometric fluorescence readouts from di-8-ANEPPS and simulations, we show that both BLA and BLA-OA complexes bind preferentially to negatively charged membranes. Importantly, membrane perturbations extend throughout the length of the acyl chain for both POPC and POPG, following the binding of BLA and the BLA-OA complexes. We also observed the clustering of POPG in the vicinity of both the BLA and BLA-OA complexes in negatively charged membranes, potentially reinforcing the specificity of interactions between the protein in BLA/BLA-OA complex and negatively charged lipid membranes.

Two types of lipid interactions with α-lactalbumin were addressed. First, OA, a basic constituent of the BAMLET/HAMLET complex, was shown to bind to peptide epitopes in both α- and β- domains of the protein, indicating an inherent plasticity in the residue level interaction of OA with BLA and that residues from almost all segments of the protein may interact with OA. Second, BLA-OA complexes were shown to engage in membrane interactions, where the protein was identified as a ligand defining the occupancy time and depth of insertion into the lipid bilayer. Importantly, a large number of the membrane interacting residues were available after the formation of BLA-OA complex, indicating that the binding to oleic acid does not inhibit membrane interactions of the lipid binding residues in BLA. Our normalized occupancy maps show that the residues are present in close proximity to the lipid chains, in BLA alone and lie predominantly in the α-domain, in the complex with OA, with some residues in the β-domain. Particularly, residues in the C-terminal part of BLA, alone and in complex with OA, showed relatively high occupancy time around lipid head groups. Furthermore, experiments in tumor cells revealed an enhanced uptake of peptide fragments mapping the C-terminal (P10, P11, P12) region of α-LA, corroborating the results from occupancy maps.

In this work, we also characterized the BLA-OA complex using tryptophan fluorescence and simulations. Our results suggest a highly dynamic and plastic interaction of OA with various residues of BLA, which might lead to a more unfolded molten-globule conformation (Figs 1, S1 and S2). This is supported by the enhanced tryptophan fluorescence observed upon addition of OA relative to BLA, suggesting release of quenching upon further unfolding.

Negatively charged lipids are more abundant in the outer leaflet of plasma membranes in various cancer cells^49^,^50^,^51^. Such clusters of negatively charged lipids have been reported as molecular beacons for the action of many important proteins such as syntaxin and synaptotagmin^52^,^53^. Targeting such lipids might provide an extracellular ligand such as BLA-OA with a recognition platform, independent of traditional protein receptor specificities^39^. This view is supported by the formation of negatively charged lipids clusters, associated with BLA/BLA-OA, reminiscent of the microscopic membrane foci of upstream Ras signaling cascades formed, in tumor cells treated with HAMLET^54^. In addition, recent studies have shown that the creation of specific membrane perturbations allows HAMLET to target several Ras family proteins in their membrane-bound, active state and to dysregulate oncogene-dependent functions of these GTPases, as well as other kinases, to which tumor cells are addicted^54^.

Early studies suggested that the HLA/BLA-OA complexes possess unique novel properties not found in its constituents. Partially unfolded α-lactalbumin lacks tumoricidal activity and that OA alone does not reproduce HAMLET’s effects, at concentrations present in the complex. The extent to which the OA contributes to the tumor cell death in response to HAMLET/BAMLET has been extensively debated. A group of studies have concluded that the toxicity of the complex is dependent on OA and α-LA acts as a carrier to increase the water solubility of OA^16^,^20^,^55^. These contradictions reflect the use of production methods to generate protein-lipid complexes that are different from HAMLET and less well characterized, structurally and functionally. Based on the present study, we conclude that the BLA-OA complex achieves specificity for tumor cell membranes through protein interactions that are maintained even in the lipid complex, in the presence of OA.

## Conclusions

To summarize, we obtained molecular insights into the interaction of BLA/BLA-OA complex with model membranes. We characterized the folding state of BLA-OA complex using tryptophan fluorescence and resolved residue-specific interactions of BLA with OA using coarse-grained and atomistic molecular dynamics simulation. We integrated membrane-binding data from a voltage-sensitive membrane fluorescent probe (di-8-ANEPPS) and coarse-grained simulations to demonstrate the differential interaction of the BLA-OA complex with negatively charged membranes. Furthermore, we identified amino acid residues of BLA and BLA-OA complex as determinants of these membrane interactions using coarse-grained simulations. Biological relevance was corroborated by uptake of the corresponding α-LA peptides across tumor cell membranes, alone and in combination with OA. Interestingly, we observed a characteristic clustering of POPG around the site of protein binding in case of BLA/BLA-OA complex monitored by the analysis of the two-dimensional density distribution of different lipid components following the stable binding of BLA and BLA-OA complex to the negatively-charged bilayer in coarse-grained simulations. To the best of our knowledge, our results constitute the first report comprehensively exploring the lipid-protein interaction of the partially disordered α-lactalbumin and its complex with OA in particular. The results suggest that the α-LA component of these cytotoxic complexes confers specificity for tumor cell membranes through protein interactions that are maintained even in the lipid complex, in the presence of OA.

## Experimental Section

### Material

1-Palmitoyl-2-oleoyl-*sn*-glycero-3-phosphocholine (POPC) and 1-palmitoyl-2-oleoyl-*sn*-glycero-3-phosphatidylglycerol (POPG) were purchased from Avanti Polar Lipids (Alabaster, AL). Calcium depleted BLA (*apo*-BLA), cholesterol, DMPC, uranyl acetate, penicillin, streptomycin, gentamycin sulfate and OA were from Sigma Chemical Co. (St. Louis, MO). DMEM/F-12 (Dulbecco’s Modified Eagle Medium/Nutrient Mixture F-12, 1:1), DMEM and fetal calf serum were from Gibco/Life Technologies (Grand Island, NY). Lipids were checked for purity by thin layer chromatography on silica gel precoated plates obtained from Merck (Darmstadt, Germany) in chloroform/methanol/water (65:35:5, v/v/v) and were found to give a single spot in all cases when visualized upon charring with a solution containing cupric sulfate (10%, w/v) and phosphoric acid (8%, v/v) at 150 °C^56^. Concentration of lipids were determined by phosphate assay subsequent to total digestion by perchloric acid^57^. 1,2-dimyristoyl-*sn*-glycero-3-phosphocholine (DMPC) was used as an internal standard. The concentration of BLA in aqueous solution was calculated from its molar extinction coefficient (ε) of 28,540 M^−1^cm^−1^ at 280 nm^58^. The concentration of the stock solution of di-8-ANEPPS in methanol was estimated from its molar absorption coefficient of 37,000 M^−1^cm^−1^ at 498 nm^59^. All other chemicals used were of the highest purity available. Solvents used were of spectroscopic grade. Water was purified through a Millipore (Bedford, MA) Milli-Q system and used throughout.

### Sample Preparation

There are two main methods of preparing α-LA-OA complexes. The initially reported method to prepare HAMLET involves running human α-LA through an OA-conditioned anion exchange column^27^,^28^. Using this method, complexes between OA and α-LA from various species were shown to kill to tumor cells, including BLA^29^. A second method involves mixing of α-LA and OA in solution. Complexes formed by this method also have cytotoxic properties^18^,^19^,^20^,^21^,^26^.

BLA-OA complex was prepared by a modification of the simple mixing method^18^,^19^,^20^,^21^,^26^ wherein OA (600 μM) dissolved in 10 mM phosphate buffer (extruded through 200 nm filter) was added to *apo*-BLA (60 μM) prepared in 10 mM sodium phosphate (pH 7.4). In this paper, wherever BLA will be referred would indicate the *apo*-form. For BLA-OA-membrane interaction, experiments were performed using large unilamellar vesicles (LUVs) of 100 nm diameter composed of POPC and 70% POPC/30% POPG (mol/mol). Total lipid and di-8-ANEPPS concentrations were 200 and 4 μM, respectively in each sample. For LUV preparation, stock solution of lipids of varying composition (total lipid stock of 5 μmol) were mixed well and dried under a stream of nitrogen while being warmed gently (~35 °C). After further drying under high vacuum for at least 3 h, the lipid mixture was hydrated (swelled) by addition of 5 mL of 10 mM phosphate buffer (pH 7.4), and each sample was vortexed for 3 min to uniformly disperse the lipids and form homogeneous multilamellar vesicles. LUVs of 100 nm diameter were prepared by the extrusion technique using an Avestin Liposofast Extruder (Ottawa, Ontario, Canada) as previously described^60^. Briefly, the multilamellar vesicles were freeze-thawed five times using liquid nitrogen to ensure solute equilibration between trapped and bulk solutions and then extruded through polycarbonate filters (pore diameter of 100 nm) mounted on an extruder fitted with Hamilton syringes (Hamilton Company, Reno, NV). The samples were subjected to 11 passes through the polycarbonate filters to give the final LUV suspension. The samples were diluted from 1 mM stock concentration to 200 μM for each sample. Appropriate volumes of BLA-OA complex were added to the LUVs of various composition. Samples were incubated in dark for 12 h at room temperature (~23 °C) for equilibration before measuring fluorescence. All experiments were done with multiple sets of samples at room temperature (~23 °C).

### Circular Dichroism (CD) and Tryptophan Fluorescence Measurements

Details of CD and tryptophan fluorescence measurements for BLA in comparison to BLA-OA complex are provided in Supporting Information. The tryptophan fluorescence measurements were carried out 295 nm excitation.

### Binding Studies utilizing di-8-ANEPPS Fluorescence

Fluorescence excitation spectra measurement were carried out as described earlier^5^. Details are provided in Supporting Information.

### Cellular assays for peptide uptake

Cellular assays for peptide uptake were performed as described earlier^32^. The designed peptides were custom synthesized by Mimotopes (Clayton, Australia). The peptides were synthesized using the mild Fmoc chemistry method. For biotinyalation of the peptides, an aminohexanoic acid spacer was added to ensure adequate separation between the biotin and the peptide moiety. The primary sequence follows the residue numbering in human α-LA. A library of biotinylated peptides from the N- to C-termini of α-LA, each 15 amino acids long with 5 amino acid overlaps were generated for the cellular assays.

For cellular uptake assays, A549 human lung carcinoma cell line (obtained from ATCC, Manassas, VA, USA) was cultured in RPMI-1640 with non-essential amino acids (1:100), 1 mM sodium pyruvate (all from PAA, Pasching, Austria), 50 mg/ml gentamicin (Gibco, Paisley, UK) and 5% fetal calf serum (FCS) at 37 °C, 5% CO_2_. The stock peptide-OA complex was freshly prepared by simply mixing 5 mM peptide and 3.5 mM sodium oleate at room temperature. These cells (5 × 10^5^ cells/ml) in suspension were incubated with either peptide alone (105 μM) or peptide-oleate mixtures (105 μM peptide, 175 μM oleate) in serum-free RPMI-1640 for 1 hour at 37 °C, 5% CO_2_ after which cells were fixed (4% paraformaldehyde) and permeabilized (0.25% Triton X-100, 5% FCS in phosphate-buffered saline). Peptides were visualized by incubation with streptavidin-Alexa568 (Molecular Probes). Images were captured on a LSM510 META confocal microscope (Carl Zeiss, Jena, Germany) with pinhole settings corresponding to one airy unit. Quantification of fluorescence intensities was performed with the ImageJ software.

### MD Simulations

#### System setup

All simulations and analysis were performed using the GROMACS simulation package, version 4.5.5^61^. VMD was used for visualization and rendering images^62^.

Crystal structure of the *apo* form of BLA (PDB:1F6R) was used for this study. The structure was solvated, charge neutralized, energy minimized and equilibrated for 100 ps under position restraints, followed by a short simulation run (1 ns) without any position restraints. The resulting structure was used as the starting conformation for atomistic simulation. Additionally this conformation was mapped to its CG representation for carrying out simulations. The CG systems were represented using the MARTINI force field (version 2.1)^63^,^64^.

A single OA molecule was randomly positioned into the solvated BLA system at a distance ~2 nm from protein surface for atomistic and CG simulations. The resulting system was then energy minimized and equilibrated. Ten independent atomistic and CG simulations were carried out with different initial velocities. The atomistic and CG simulations were extended till 0.5 and 1 μs, respectively. Details regarding the system composition and initial conformation are given in Table S5 in Supporting Information.

In order to investigate binding positions of subsequently added OA molecules, single OA bound BLA conformation obtained at the end of CG simulation was taken as the starting structure. A new OA molecule was randomly positioned at a distance of ~2 nm from the BLA-OA complex. The system was then energy minimized and simulated for 1 μs. Similar procedure was repeated for addition of six subsequent OA molecules, with the BLA-OA bound complex of the previous simulation serving as the starting structure for subsequent addition. Five independent simulations were carried out for each BLA-OA complex for a period of 1 μs, starting with different initial velocities.

Bilayers of the composition POPC (PC) and 70% POPC/30% POPG (PC/PG) were self assembled from an initial conformation of randomly placed POPC, POPG and water beads. The bilayers formed in the simulation were equilibrated for 30 ns to ensure uniform distribution of lipids. Details regarding the number of lipids and water used for each system are given in Table S6 in Supporting Information. A single copy of BLA (alone or in complex with six OA molecules) was placed at a minimum distance of at least 2 nm from the bilayer surface. Simulations were carried out for a period of 10 μs each. Five independent simulations were carried out for each system, starting with different initial velocities.

#### Atomistic simulation parameters

OPLS-AA forcefield and SPC water model was employed for atomistic simulation. The OA forcefield (neutral) was obtained by modification of the oleoyl chain of POPC^65^. A cut-off of 1 nm for electrostatic and *van der Waals* interactions was used for calculating short range non-bonded interactions while Particle-Mesh Ewald (PME)^66^ with cubic interpolation was used for long-range electrostatistics. Steepest descent algorithm was employed for energy minimization procedure. Temperature of all groups (BLA, OA, ion and water) was maintained at 333 K using V-rescale thermostat^41^ with a coupling constant of 0.1 ps. Isotropic pressure was maintained using Parrinello-Rahman barostat^42^ with 1 bar independently in the X, Y and Z planes with a coupling constant of 2.0 ps and a compressibility of 4.5 × 10^−5^ bar^−1^. LINCS algorithm was used to constrain all bonds^43^. Simulations were carried out using leap-frog algorithm with an integration time step of 2 fs. Neighbor list was updated every 10 fs with a cut-off at 1.0 nm and periodic boundary conditions maintained along x, y and z axes.

#### Coarse-Grained Simulations

MARTINI coarse grain models and force-field parameters (version 2.1) were used for CG simulations. OA forcefield was obtained by modification of the oleyl chain of POPC. P3 MARTINI bead type was used to model the headgroup (-COOH) of OA. Lennard-Jones potential was gradually shifted to 0 between 0.9–1.2 nm while electrostatic interactions were shifted to 0 in the range 0–1.2 nm. A dielectric screening of 15 was used for solvent. Steepest descent algorithm was employed for energy minimization procedure. Temperature for BLA, OA and water was maintained at 300 K by weakly coupling to Berendsen thermostat^44^ with a coupling constant of 0.1 ps. Isotropic pressure was maintained using Berendsen barostat^44^ at 1 bar independently in the X,Y and Z planes with a coupling constant of 0.5 ps and a compressibility of 3 × 10^−5^ bar^−1^. Simulations was carried out with an integration time step of 20 fs. For membrane simulations, temperature for BLA, OA, POPC and POPG and water were maintained at 300 K using V-rescale thermostat^41^ with a coupling constant of 0.1 ps. Semi-isotropic pressure was maintained using the Berendsen barostat algorithm with a pressure of 1 bar independently in the plane of the membrane and perpendicular to the membrane, a coupling constant of 0.5 ps, and a compressibility of 3 × 10^−5^ bar^−1^. Neighbor list was updated every 0.2 ps with a cut-off at 1.2 nm and periodic boundary conditions maintained along x, y and z axes.

#### Analysis of the binding sites for OA on BLA surface

We refer to the time period, starting from the instance OA made first contact with BLA, as the OA-bound period. A cut-off distance of 0.4 nm and 0.55 nm were chosen for atomistic and CG simulations respectively to quantitate direct interaction between BLA and OA. Binding sites for OA on BLA surface were determined by calculating the total occupancy period of OA around each amino acid residue of BLA over the total OA-bound (occupancy) period. Total occupancy period is defined as the time period over which BLA shows direct interaction with OA. This normalized value was averaged over all ten simulation sets.

#### Analysis of the binding sites of BLA/BLA-OA complex with the lipid bilayer

The total occupancy time of BLA/BLA-OA complex residues around the CG beads of the phospholipids representing GL2 and subsequent beads of the palmitoyl chain (C1A, C2A,C3A and C4A) were calculated and normalized to the membrane-bound period. The normalized values were averaged over the five sets of simulations. The membrane-bound period corresponds to the simulation period starting from the instance BLA/BLA-OA complex makes first stable contact with the lipid bilayer. The cut-off value for the interaction was set at 0.53 nm.

#### Analysis of the bilayer physical properties

To analyze the effect of binding of BLA/BLA-OA complex on bilayer properties, we generated two-dimensional plots for bilayer thickness, lipid order-parameter, lipid density and water density over the entire membrane-bound period for bilayers of different composition. For these calculations, the protein was centered in the bilayer and its translation motion removed. Bilayer thickness was calculated as the average distance between the PO4 beads of the two leaflets using previously developed analysis tools. One-dimensional lipid order parameter (P_2_) for POPC and POPG in the bilayer systems were determined from the equation:


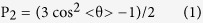


for each consecutive bonds in the coarse-grain lipids, where θ denotes the angle between the bond vector connecting consecutive beads and bilayer normal. A P_2_ value of 1, −0.5 and 0 indicate perfect alignment, perfect anti-alignment and random orientation, respectively. The density distribution for lipids were calculated using previously developed analysis tools^45^.

## Additional Information

**How to cite this article**: Chaudhuri, A. *et al*. Protein-dependent Membrane Interaction of A Partially Disordered Protein Complex with Oleic Acid: Implications for Cancer Lipidomics. *Sci. Rep.* **6**, 35015; doi: 10.1038/srep35015 (2016).

## Supplementary Material

Supplementary Information
