# Supplementary material for: Protein-dependent Membrane Interaction of A Partially Disordered Protein Complex with Oleic Acid: Implications for Cancer Lipidomics
Source: Sci Rep. 2016 Oct 12;6:35015. doi: 10.1038/srep35015 (PMC5059734; doi:10.1038/srep35015)
Supplement: Supplementary Information [file srep35015-s1.pdf]

# SUPPORTING INFORMATION

## Protein-dependent Membrane Interaction of A Partially Disordered Protein Complex with Oleic Acid: Implications for Cancer Lipidomics

Arunima Chaudhuri,[§,†,$] Xavier Prasanna,[‡,†] Priyanka Agiru,[§] Hirak Chakraborty,[§,¶] Anna Rydström,[ǂ] James C.S. Ho,[#] Catharina Svanborg,[ǂ] Durba Sengupta[‡,*] and Amitabha Chattopadhyay[§,*]

[§]CSIR-Centre for Cellular and Molecular Biology, Uppal Road, Hyderabad 500 007, India; [‡]CSIR-National Chemical Laboratory, Pune 411 008, India; [ǂ]Department of Microbiology, Immunology and Glycobiology (MIG), Institute of Laboratory Medicine, Lund University, Lund, Sweden; [#]Centre for Biomimetic Sensor Science, School of Materials Science & Engineering, Nanyang Technological University, Singapore 637553

[†]Equal contribution

*Address correspondence to Amitabha Chattopadhyay, Tel: +91-40-2719-2578, Fax: +91-2716-0311, E-mail: amit@ccmb.res.in; or Durba Sengupta, E-mail: d.sengupta@ncl.res.in

[$]Present address: Department of Cell Biology, Yale University, New Haven, CT 06511;
[¶]Present address: School of Chemistry, Sambalpur University, Burla, Odisha 768 019, India

**Tryptophan Fluorescence Measurements.** Steady state fluorescence measurements of BLA and BLA-OA complex were performed with a Hitachi F-7000 spectrofluorometer (Tokyo, Japan) using 1 cm path length quartz cuvettes. Excitation and emission slits with a nominal bandpass of 2.5 nm were used for all measurements. Background intensities of samples in which the peptide was omitted were subtracted from each sample spectrum to cancel out any contribution due to the solvent Raman peak and other scattering artifacts. The spectral shifts obtained with different sets of samples were identical in most cases, or were within ± 1 nm of the ones reported. Fluorescence anisotropy measurements were performed at room temperature (~25 °C) using a Hitachi polarization accessory.

**Time-Resolved Fluorescence Measurements.** Fluorescence lifetimes were calculated from time-resolved fluorescence intensity decays using IBH 5000F NanoLED equipment (Horiba Jobin Yvon, Edison, NJ) with DataStation software in the time-correlated single photon counting (TCSPC) mode. A pulsed light emitting diode (LED) (NanoLED-17) was used as the excitation source. This LED generates optical pulse at 294 nm with pulse duration less than 750 ps, and is run at 1 MHz repetition rate. The LED profile (instrument response function) was measured at the excitation wavelength using Ludox (colloidal silica) as the scatterer. In order to optimize the signal to noise ratio, 10,000 photon counts were collected in the peak channel. All experiments were performed using emission slits with a bandpass of 6 nm or less. The sample and the scatterer were alternated after every 5% acquisition to ensure compensation for any shape and timing drifts that could occur during the period of data collection. This arrangement also prevents prolonged exposure of the sample to the excitation beam, thereby avoiding any possible photodamage to the fluorophore. Data were stored and analyzed using DAS 6.2 software (Horiba Jobin Yvon, Edison, NJ). Fluorescence intensity decay curves so obtained were deconvoluted with the instrument response function and analyzed as a sum of exponential terms:

$$F(t) = \Sigma_i \alpha_i \exp(- t/\tau_i) \qquad (1)$$

where F(t) is the fluorescence intensity at time t and $\alpha_i$ is a pre-exponential factor representing the fractional contribution to the time-resolved decay of the component with a lifetime $\tau_i$ such that $\Sigma_i \alpha_i = 1$.

Decay parameters were recovered using a nonlinear least squares iterative fitting procedure based on the Marquardt algorithm.[3] The program also includes statistical and plotting subroutine packages.[4] The goodness of the fit of a given set of observed data and the chosen function was evaluated by the $\chi^2$ ratio, the weighted residuals[5], and the autocorrelation function of the weighted residuals.[6] A fit was considered acceptable when plots of the weighted residuals and the autocorrelation function showed random deviation about zero with a minimum $\chi^2$ value not more than 1.4. Intensity-averaged mean lifetimes ($\langle\tau\rangle$) for triexponential decays of fluorescence were calculated from the decay times and pre-exponential factors using the following equation:

$$\langle\tau\rangle = \frac{\alpha_1\tau_1^2 + \alpha_2\tau_2^2 + \alpha_3\tau_3^2}{\alpha_1\tau_1 + \alpha_2\tau_2 + \alpha_3\tau_3} \qquad (2)$$

***Circular Dichroism (CD) Measurements.*** CD measurements were carried out at room temperature (~25 °C) with a JASCO J-815 spectropolarimeter (Tokyo, Japan) calibrated with (+)-10-camphorsulfonic acid.[1] Spectra were scanned in a quartz optical cell with a path length of 0.1 cm for extended far-UV and 1 cm for near-UV range, and recorded in 0.5 nm wavelength increments and band width of 2 nm. For monitoring changes in secondary and tertiary structures, spectra were scanned from 200 to 250 nm in the extended far-UV range, and from 250 to 300 nm in the near-UV range, respectively. The scan rate was 50 nm/min and each spectrum was recorded as the average of 8 scans with a full scale sensitivity of 100 mdeg. Spectra were corrected for background by subtraction of appropriate blanks. Data are represented as mean residue ellipticities and calculated using the equation:

$$[\theta] = \theta_{obs} / (10Cl) \qquad (3)$$

where $\theta_{obs}$ is the observed ellipticity in mdeg, l is the path length in cm, and C is the concentration of peptide bonds in mol/L.

**Figure S1**
Chaudhuri *et al.*

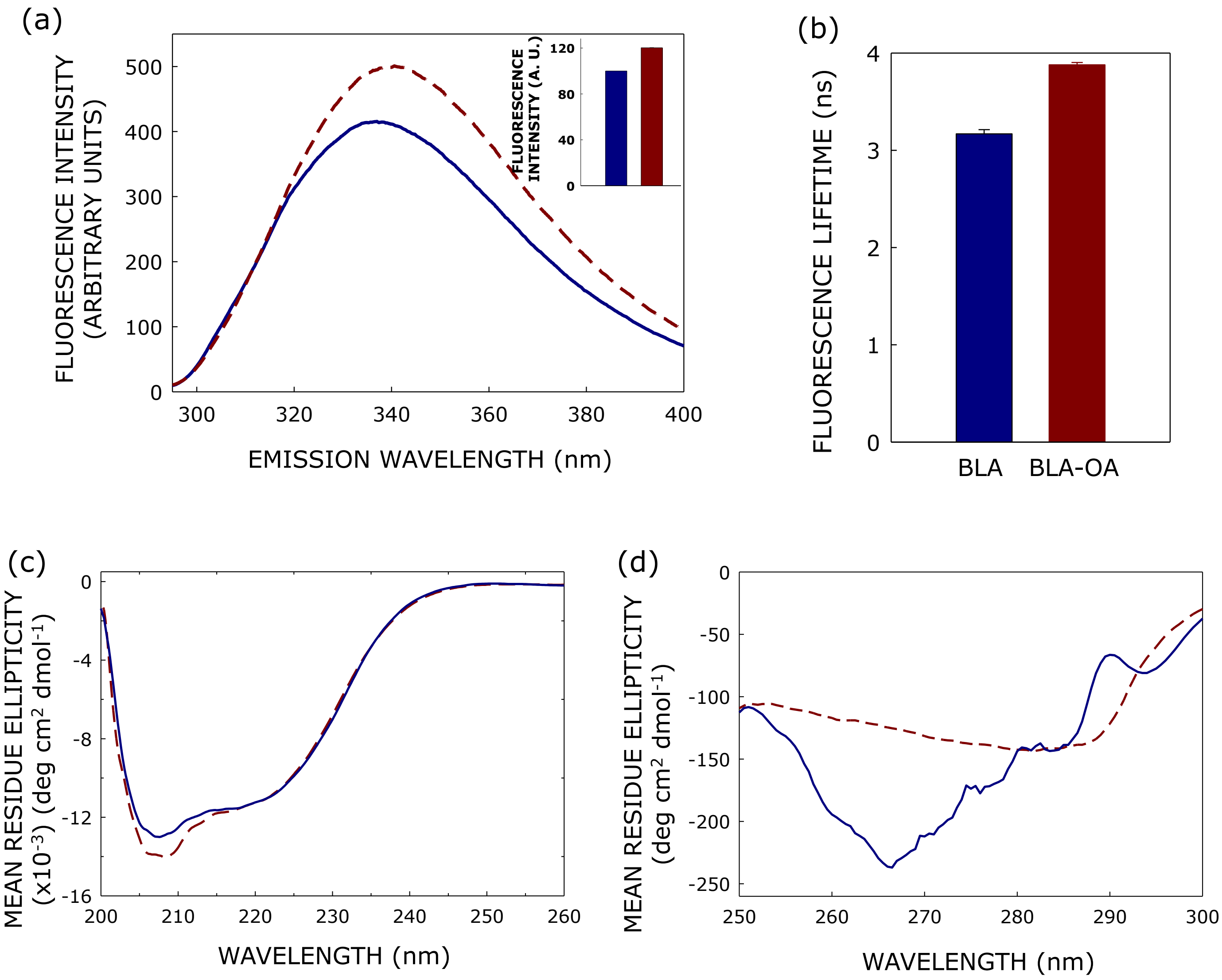


**FIGURE S1**. (a) Tryptophan fluorescence emission spectra of *apo*-BLA (blue, —) and *apo*-BLA-OA complex (maroon, - - -) complex in pH 7.4 buffer. The excitation wavelength was 280 nm in both cases. The inset shows relative fluorescence intensities of *apo*-BLA (left) and *apo*-BLA-OA complex (right). (b) Tryptophan fluorescence lifetime of *apo*-BLA and *apo*-BLA-OA complex are shown. The excitation wavelength was 295 nm and emission monitored at 335 nm in both cases. Data shown are means ± S.E. of at least three independent measurements. Representative (c) extended far-UV and (d) near-UV CD spectra of *apo*-BLA (—) and *apo*-BLA-OA complex (- - -) in buffer. All other conditions are same as in Figure S1. See Materials and methods for further details. The concentration of *apo*-BLA was 60 μM and *apo*-BLA: OA ratio was 1:10 (mol/mol) in 10 mM phosphate buffer. See Materials and methods for further details.

**Figure S2**
Chaudhuri *et al.*

(a)

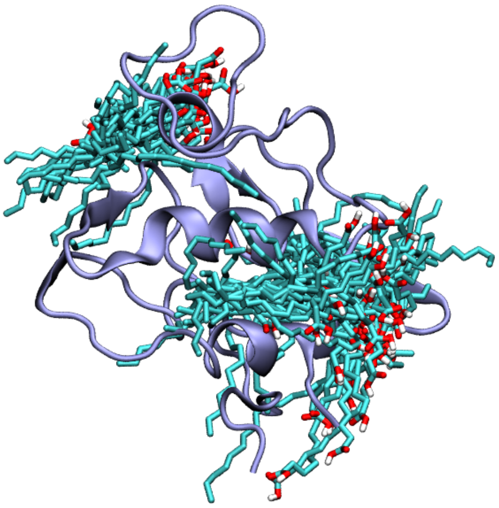

(b)

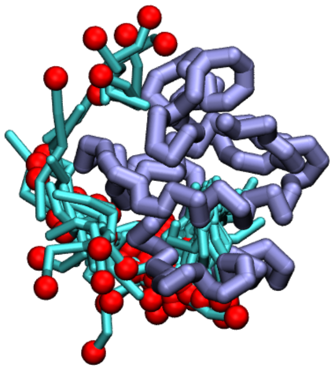

**Figure S2.** Representative snapshots showing few of the sites sampled by a single OA molecules on BLA obtained from (a) atomistic and (b) CG simulations. BLA is shown in ice blue; OA headgroup and tail are shown in red and cyan, respectively. The figure depicts the dynamics of OA tail during interaction with BLA and the plasticity involved in the contact sites. For atomistic simulation, each OA molecule shown in the figure represents the position of the OA recorded at a time interval of 4 ns, and sampling done for a total period of 400 ns. For CG simulations, the snapshot represents a sampling time of 100 ns with an interval of 1 ns. See Materials and methods for further details.

**Figure S3**
Chaudhuri *et al.*

(a)

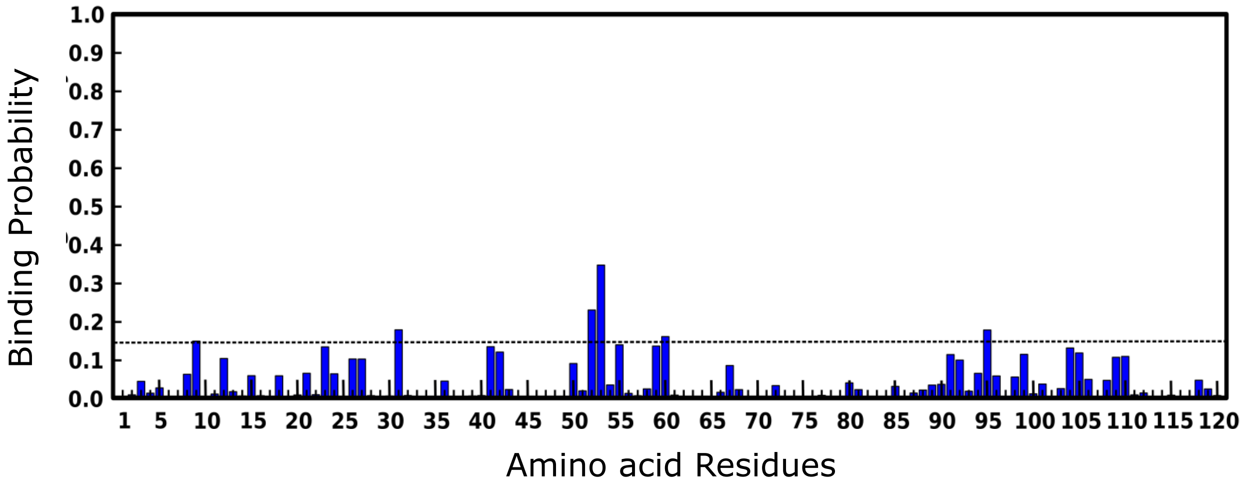


(b)

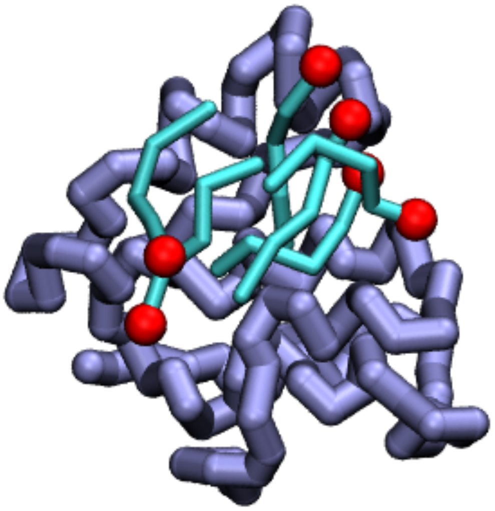

**Figure S3.** Binding probability of sequentially added OA molecules (total of six) with the residues of BLA for CG simulations is shown. The binding probability for each subsequent addition has been normalized over the total bound period and averaged over ten simulations are shown. The residues on and above the dotted line represent the most probable residues interacting with OA molecules. (b) Schematic representation showing the binding site of the six consecutively added OA molecules is show. The color coding of BLA and OA is same as in figure S1. See Materials and methods for further details.

***Binding Studies utilizing di-8-ANEPPS Fluorescence.*** Fluorescence excitation spectra were recorded using a Hitachi F-7000 spectrofluorometer (Tokyo, Japan) with 1 cm path length quartz cuvette. Emission wavelength was fixed at 670 nm. Excitation and emission slits with a nominal bandpass of 2.5 nm were used for all measurements. Background intensities of samples were subtracted from each sample to cancel any contribution due to the solvent Raman peak and other scattering artifacts. The fluorescence ratio (R), defined as the ratio of fluorescence intensities at an excitation wavelength of 455 nm to that at 525 nm (emission at 670 nm in both cases) which is a measure of membrane dipole potential, was calculated from di-8-ANEPPS excitation spectra.[7-9] The choice of the emission wavelength (670 nm) at the red edge of the spectrum has previously been shown to rule out membrane fluidity effects.[7] Normalized fluorescence ratio $R_{455/525}$ was obtained by dividing the fluorescence ratio obtained for various concentrations of BLA in presence of membranes with the fluorescence ratio obtained with only the probe (di-8-ANEPPS) in the membrane. The normalized $R_{455/525}$ for each concentration of BLA was plotted and fitted to a simple hyperbolic function utilizing Sigma Plot (Systat Software Inc., San Jose, CA) using the following equation:

$$\text{Normalized } R_{455/525} = 1 + (B_{max}\,[BLA])/(K_d + [BLA]) \qquad (5)$$

which describes a single binding site model and where $K_d$ is the apparent dissociation constant and $B_{max}$ corresponds to the maximum value of $R_{455/525}$.

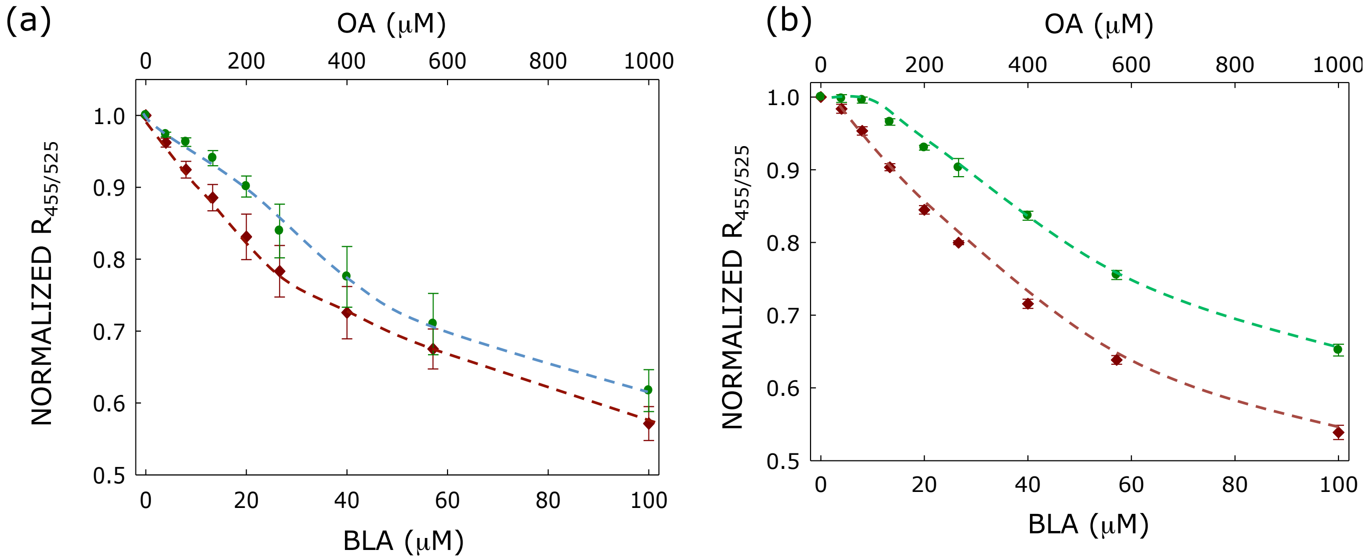


**Figure S4.** Normalized fluorescence ratio ($R_{455/525}$) derived from the excitation spectra of di-8-ANEPPS in membranes of various composition with increasing *apo*-BLA-OA complex (◆, BLA and OA concentration in bottom and top X-axes, respectively) and OA (●, top X-axis) are shown for (a) POPC and (b) 30%POPG/70%POPC. The normalized $R_{455/525}$ shown are means ± S.E. of at least three independent measurements. The concentration of phospholipids and di-8-ANEPPS were 200 and 4 μM, respectively. Lines joining the data points are provided merely as viewing guides. See Materials and methods for further details.

**Figure S5**

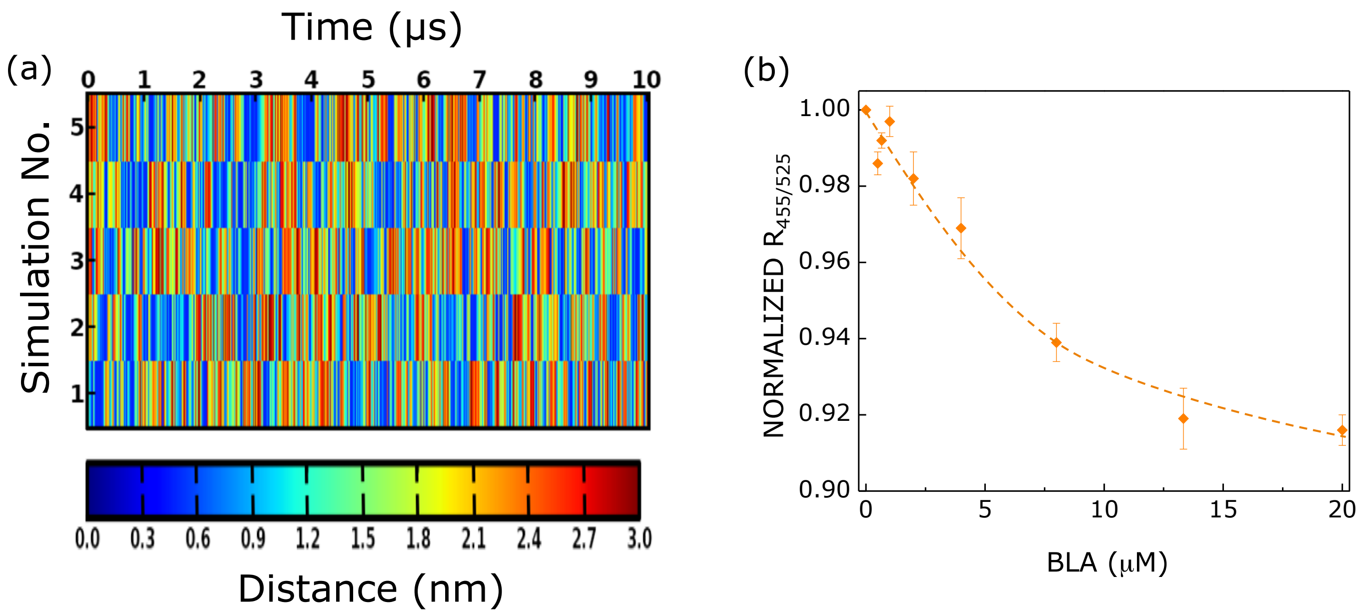


**FIGURE S5**. Differential interaction of BLA with zwitterionic membranes using simulation and experiment. (a) Time course of interaction of BLA with PC bilayers of using CG simulation. The minimum distance between BLA and lipid (defined as the closest distance of approach between CG beads of BLA and lipid) during the course of five simulations are plotted for zwitterionic bilayer. The color bar below refers to the scale for minimum distance of interaction. Blue regions represent close contacts relative to red regions which indicate larger distance between BLA and the bilayer. See Materials and methods for further details. (b,d,f) Membrane interaction of *apo*-BLA monitored using the fluorescence of the potential-sensitive probe di-8-ANEPPS. Binding plot of *apo*-BLA with POPC (◆,b) membranes utilizing di-8-ANEPPS dual ratiometric fluorescence approach. The concentration of phospholipids and di-8-ANEPPS were 200 and 4 μM, respectively. Lines joining the data points are provided merely as viewing guides. Data adapted and modified from ref.5.

**Figure S6**
Chaudhuri *et al.*

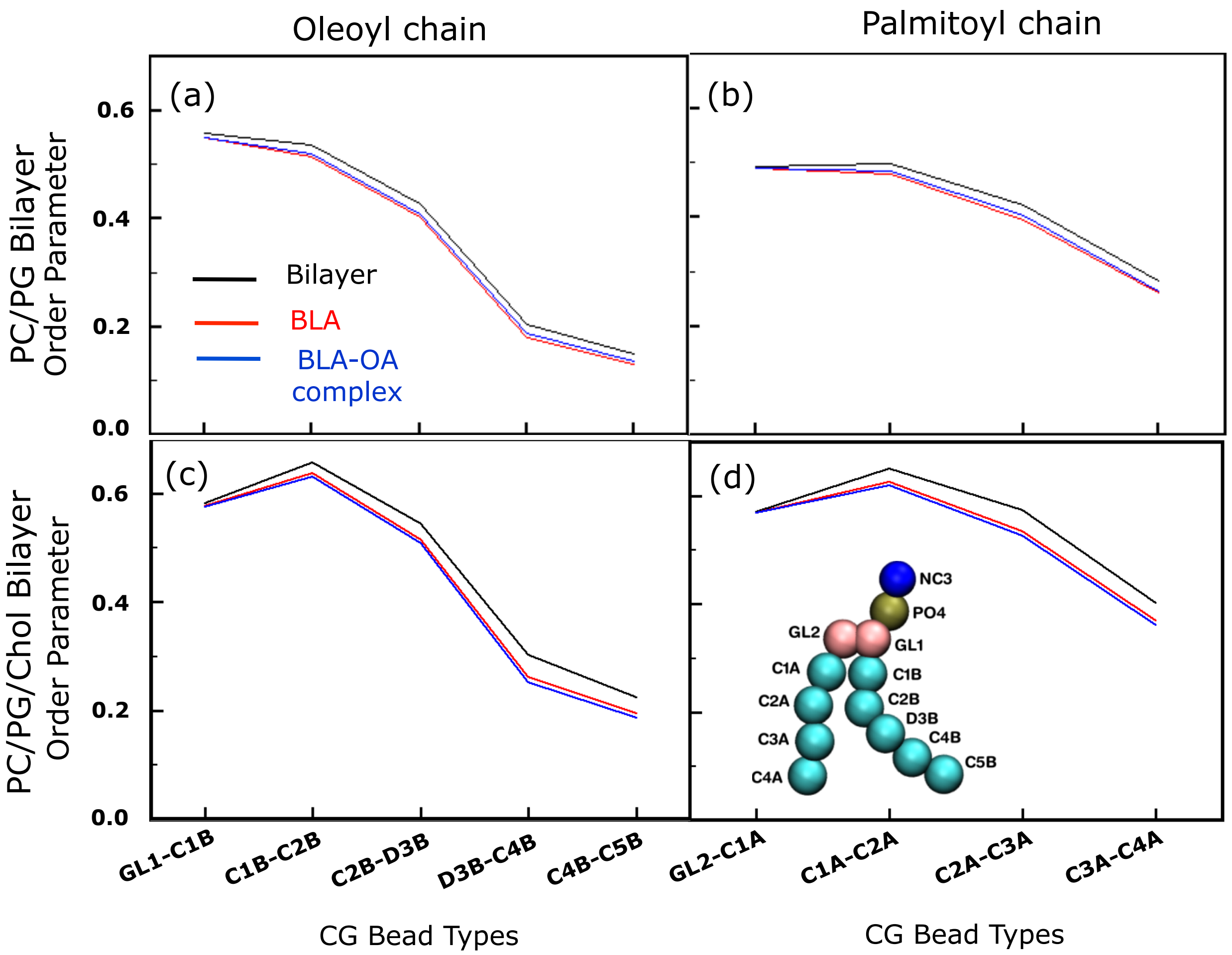


**Figure S6**. One-dimensional order parameter of POPC in PC/PG (a,b) and PC/PG/Chol (c,d) bilayers in the presence of BLA and BLA-OA complex. The order parameters for the oleoyl (a,c) and palmitoyl (b,d) chains of POPC in the absence (black line) and presence of BLA (red line) and BLA-OA (blue line) are shown. Schematic CG representation of POPC is given as an inset of Figure S5D. See Methods for other details.

| **Table S1**<br><br>**Residues of BLA interacting with OA: Comparing experimental and simulation results** | | | | | |
|---|---|---|---|---|---|
| Regions | Experimental Results | | | Simulation Results of single OA binding (Atomistic and CG combined)[a] | Simulation Results of sequential OA binding (CG)[a] |
| | Ref.10 | Ref.11 | Ref.12 | | |
| Helix A | 8,9 | | | **8,9** | **9** |
| Helix B | 23,31,33 | | | **23**,27,**31** | **31** |
| Helix C | 86 | 86-96 | | **89,92,95,96** | **95** |
| Helix D | | | 105-109 | | |
| Other regions of α-domain | 13,16,22, 25, | 70 | 110-123 | 12 | |
| β-domain | 35,37, 42,83,84 | 39,41-52, 53-59, 60-85 | | **53** | **52**,**53,60** |

[a]The residues having high occupancy around OA are shown. The residues shown in bold are common ones between simulation and previous experimental results.

**Table S2**

**Normalized Occupancy Period of residues in BLA and BLA-OA complex around GL2 bead of palmitoyl chain of lipids[a]**

| Secondary Structure | BLA | | BLA-OA | |
|---|---|---|---|---|
| | PC/PG bilayer | PC/PG/Chol bilayer | PC/PG bilayer | PC/PG/Chol bilayer |
| Helix A | | Lys-5 | Arg-10 | |
| Helix B | | | Trp-26 | |
| Helix C | Lys-98 | Lys-98 | Lys-98 | Lys-98 |
| Helix D | Leu-105, Lys-108 | Lys-108 | Leu-105, His-107, Lys-108, Ala-109 | Leu-105, Lys-108 |
| Other regions of $\alpha$-domain | Tyr-18, Val-21, Ala-99, Ile-101, Tyr-103, Trp-104, Leu-110, Lys-114, Trp-118, Leu-119 | Lys-13, Lys-16, Tyr-18, Val-21, Ile-101, Tyr-103, Trp-104 | Lys-16, Tyr-18, Gly-20, Val-21, Ser-22, Val-99, Gly-100, Ile-101, Asn-102, Tyr-103, Trp-104, Leu-110, Cys-111, Ser-112, Lys-114, Trp-118, Leu-119 | Ile-101, Tyr-103, Trp-104, Leu-110, Lys-104 |
| $\beta$-domain | Ile-41, Val-42, Gln-43, Trp-60, | Ile-41, Val-42, Gln-43, Ile-55, Lys-58, Ile-59 | Ile-41, Val-42, Gln-43, Tyr-50, Leu-52, Gln-54, Ile-55, Lys-58, Ile-59, Pro-67, Ile-72 | Ile-41, Val-42, Gln-43, Tyr-50, Leu-52, Phe-53, Ile-55, Lys-58, Ile-59, Trp-60 |

[a]The total occupancy period of BLA residues were normalized to the total membrane-bound period and the values were averaged over five sets of simulations. Residues with values ≥ 0.25 were considered.

| **Table S3** | | | | |
|---|---|---|---|---|
| **Normalized Occupancy Period of residues in BLA and BLA-OA complex around C1A bead of palmitoyl chain of lipids[a]** | | | | |
| **Secondary Structure** | **BLA** | | **BLA-OA** | |
| | **PC/PG bilayer** | **PC/PG/Chol bilayer** | **PC/PG bilayer** | **PC/PG/Chol bilayer** |
| Helix A | | | | |
| Helix B | Phe-31 | | Trp-26 | |
| Helix C | | | | |
| Helix D | Leu-105 | | Leu-105 | Leu-105 |
| Other regions of $\alpha$-domain | Val-99, Trp-104, Leu-110, Trp-118 | Trp-118 | Val-21, Tyr-103, Trp-104, Trp-118 | Trp-104 |
| $\beta$-domain | Ile-41, Val-42, Phe-53 | Phe-53 | | Ile-41, Val-42, Leu-52, Phe-53, Ile-55, Ile-59, Trp-60 |

[a]The total occupancy period of BLA residues were normalized to the total membrane-bound period and the values were averaged over five sets of simulations. Residues with values ≥ 0.25 were considered.

| **Table S4** | | | | |
|---|---|---|---|---|
| **Normalized Occupancy Period of residues in BLA and BLA-OA complex around C2A bead of palmitoyl chain of lipids[a]** | | | | |
| Secondary Structure | BLA | | BLA-OA | |
| | PC/PG bilayer | PC/PG/Chol bilayer | PC/PG bilayer | PC/PG/Chol bilayer |
| Helix A | | | | |
| Helix B | | | | |
| Helix C | | | | |
| Helix D | Leu-105 | | | |
| Other regions of α-domain | Trp-104 | | Trp-104 | |
| β-domain | | Leu-52 | | Phe-53 |

[a]The total occupancy period of BLA residues were normalized to the total membrane-bound period and the values were averaged over five sets of simulations. Residues with values ≥ 0.25 were considered.

| **Table S5**<br><br>**Initial System conformation for BLA-OA interaction** | | | | | |
|---|---|---|---|---|---|
| **System** | **Initial minimum distance (nm)** | **No. of simulations** | **Simulation lengths (μs)** | **No. of molecules** | |
| | | | | **BLA** | **OA** |
| BLA + OA (Atomistic) | 2.62 | 10 | 0.5 | 1 | 1 |
| BLA + OA (CG) | 2.95 | 10 | 1 | 1 | 1 |
| BLA + OA[a] (CG) | ~2 | 10 | 1 | 1 | 6 |

[a] Five randomly chosen final conformation of BLA bound to a single OA were taken as the starting structure for subsequent sequential addition of OA. The sequential addition over the five randomly chosen conformations of BLA bound to single OA were performed in duplicate.

| Table S6<br><br>System composition and initial conformation for BLA/BLA-OA complex membrane interaction | | | | | | | |
|---|---|---|---|---|---|---|---|
| **System** | **Initial minimum distance (nm)** | **No. of simulations** | **Simulation lengths (μs)** | **Number of molecules** | | | |
| | | | | POPC | POPG | Chol | Water |
| BLA with PC bilayer | 2.1 | 5 | 10 | 160 | 0 | 0 | 4255 |
| BLA + OA with PC bilayer | 2.5 | 5 | 10 | 160 | 0 | 0 | 4255 |
| BLA with PC/PG bilayer | 1.9 | 5 | 10 | 112 | 48 | 0 | 4255 |
| BLA + OA with PC/PG bilayer | 2.0 | 5 | 10 | 112 | 48 | 0 | 4255 |
| BLA with PC/PG/Chol | 2.1 | 5 | 0.7 | 54 | 48 | 48 | 4255 |
| BLA + OA with PC/PG/Chol | 3.4 | 5 | 1 | 54 | 48 | 48 | 4255 |

## References


(1) Chen, G. C.; Yang, J. R. Two-point calibration of circular dichrometer with d-10-camphorsulfonic acid. *Anal. Lett*. **1977**, 10, 1195-1207.

(2) Lakowicz, J. R. *Principles of Fluorescence Spectroscopy,* 3rd ed.; Springer, New York, **2006**.

(3) Bevington, P. R. Data Reduction and Error Analysis for the Physical Sciences, McGraw-Hill, New York, **1969**.

(4) O'Connor, D. V.; Phillips, D. Time-correlated Single Photon, Academic Press, London, **1984**, pp 180−189.

(5) Lampert, R. A.; Chewter, L. A.; Phillips, D.; O'Connor, D. V. Roberts A.J., Meech S.R. Standards for nanosecond fluorescence decay time measurements. *Anal. Chem*. **1983**, 55, 68−73.

(6) Grinvald, A., Steinberg, I. Z. On the analysis of fluorescence decay kinetics by the method of least-squares. *Anal. Biochem*. **1974**, 59, 583−598.

(7) Clarke R. J.; Kane D. J. Optical detection of membrane dipole potential: avoidance of fluidity and dye-induced effects. *Biochim. Biophys. Acta* **1997**, 1323, 223-239.

(8) Matos, P. M.; Freitas, T.; Castanho, M. A. R. B.; Santos, N. C. The role of blood cell membrane lipids on the mode of action of HIV-1 fusion inhibitor sifuvirtide. *Biochem. Biophys. Res. Commun.* **2010**, 403, 270-274.

(9) Haldar, S.; Kanaparthi, R. K.; Samanta, A.; Chattopadhyay, A. Differential effect of cholesterol and its biosynthetic precursors on membrane dipole potential. *Biophys. J.* **2012**, 102, 1561-1569.

(10) Nakamura, T.; Aizawa, T.; Kariya, R.; Okada, S.; Demura, M.; Keiichi, K.; Makabe, K. Molecular mechanisms of the cytotoxicity of human $\alpha$-lactalbumin made lethal to tumor cells (HAMLET) and other protein-oleic acid complexes. *J. Biol. Chem.* **2013**, 288, 14408-14416.

(11) Casbarra, A.; Birolo, L.; Infusini, G.; Piaz, F. D.; Svensson, M.; Pucci, P., Svanborg, C.; Marino, G. Conformational analysis of HAMLET, the folding variant of human $\alpha$-lactalbumin associated with apoptosis. *Prot. Sci.* **2004**, 13, 1322-1330.

(12) Ho, C. S. J.; Rydstrom, A.; Manimekalai, M. S. S.; Svanborg, C.; Gruber, G. Low Resolution Solution Structure of HAMLET and the Importance of its Alpha-Domains in Tumoricidal Activity. *PLoS ONE* **2012**, 7, e53051.
